# Physiology of the Dental Structures

**Published:** 1873-09

**Authors:** S.P. Cutler


### ARTICLE III.

### *Physiology of the Dental Structures.*

BY S. P. CUTLER, M. D., D. D. S.

The term physiology has a wide and comprehensive scope and meaning, no less extensive than the entire animal and vegetable kingdom; not even stopping there, but ramifying extensively through the inorganic kingdom, embracing some fifteen or more of the simple elements in their binary aspect.

The comprehension of this subject in its broader sense, embraces that of animal, vegetable and comparative physiology. Vegetable physiology begins with the binary, so-called inorganic elements, consisting of earth, air and water, all of which are terrestrial. In searching for our starting point, our primordial prototypal germ cell, we fail to discover its *genesis*, or rather its *biogenesis*, as we have not been able to find its starting point; we have then to content ourselves by commencing with our cell already formed from pre-existing *protoplasm*, *bioplasm*, *nuclear matter*, *cytoblast*, *germinal matter*, or whatever name the most appropriate. Our real, not imaginary germ, when placed in the earth at a proper season, under favorable circumstances, has the power of attracting to itself certain binary compounds, as carbonic acid, water, ammonia and a few others, which already pre-exist in the germ cell, and in some way assimilating them by rearranging their binary character after decomposing them, and converting them into the nerve groups, namely: the quaternary and ternary; the first being true protoplasm of Mr Uxley and others, and the chief element of organic development, the cell builder; the latter the true cell inmate, the combustible in the furnace.

When the leaf of the plant is developed, or *evolved*, this leaf then has the faculty of decomposing, during sunlight mainly, the binaries mentioned above, and reconverting their chemical into organic compounds, *i. e.*, albumen and starch. With this albumen and starch, cells are formed and filled at the point of growth, constituting formed material of the plant. During this stage of growth and development, the evolution of organic out of inorganic elements, constitutes *vegetable physiology*. After this process is completed, physiology no longer in a strict sense prevails, as the dynamical forces of vegetable growths have ceased, or been converted into a state of hybernation; the object having been accomplished, the work having been done, the materials having been worked up into structures, *i. e.* organic. These structures are of service as storehouses for future building purposes, granaries where materials may be deposited, carried in and out as occasion may require, *ad libitum*.

The plant then is a connecting link between the inorganic and the animal kingdoms; the plant coming from the inorganic direct, and the animal direct from the vegetable. The inorganic kingdom exists prior to, and independent of the vegetable; the plant independent and prior to the animal, but not independent of the mineral, nor prior to it in either case.

All the forces that evolve the plant, must necessarily have pre-existed in the inorganic elements, both in their simple uncombined state, also in their binary condition, otherwise the plant must be regarded as super-terrestrial, or *miraculous* in its advent and continuance on earth. But, reasoning from analogy, and a scientific point of view, we must necessarily adopt the former conclusion—that is the *terrestrial aspect*.

All the minerals found in plants and animals are taken up, in solution from the soil by the *spongiale rootletts;* all the gases by the leaves through their *stomata* or *spiracles*, and there the most wonderful changes take place, where the minerals in solution in water meet the ærial gases, and there undergo mutual decomposition and recombination, the carbon in the carbonic acid retained and appropriated, it being the chief solid ingredient in the series. This change is mainly determined by an *ultra terrestrial* factor, being no less than the ultra spectral solar ray, *i. e*, the actinic or chemical, non-luminous and non-calorific ray; this effect is sometimes called actinism. Lime and silica are used in some plants in structural development, as in the outer covering of reeds and grasses. Lime is used in animals, in bone formation.

All the above processes take place mainly during the summer season and cease during cold weather; not so with the higher animals; their dynamical forces cannot be transformed to static force and live; the dynamic condition must continue, or physiology must permanently cease to exist.

If the sap of plants becomes too much chilled, it ceases to circulate; if the blood in warm blooded animals becomes too much chilled, it ceases to circulate, and the animal dies; not so with the cold blooded animal and plant. Some plants, the annuals for example, mature in a season, while others, as most of the perenials, have no definite limitation, and may live and grow well on to a millennium, if not destroyed by man or wearing of the elements. Some animals have but a short lived career, and almost all have somewhat fixed limits, or limits that are not much transcended; other animals, such as some of the giant pachyderms, and some of the cetaceans that continue to grow larger like trees, live from century to century—to an indefinite age.

Nutrition and growth of plants comes directly from the binary elements above named, as means, not as ends—there being no recognized object or aim, only acting in conformity to direction of forces acting on matter.

Animals as a rule, cannot make nutrition direct from the binary elements, which is the simplest and most stable of the whole chemical series of groupings, the plant forces being superior, overcome the binaries and rearrange the elements according to higher laws of organic forces or vegetable vitality. All higher animals on the other hand, derive their nutrition already compounded from the plant, though an assimilatory process has to be gone through with, and certain changes brought about, before these ends are attained. Though unlike the plant, an entire breaking up and recompounding does not take place, only a rearrangement of the same constituents, without any complete decompositions and new types, as in plant nutrition.

This preparation or assimilation takes place after food is taken into the digestive apparatus of animals, and there undergoes proper changes in mass, to enter the blood for its final career in the vital roll of life, which means molecular and physical motion. After animal life has once begun, it has to be sustained and perpetuated by constant additions of elements, of force, as food and oxygen, which enter from different channels, and meet in common in the blood and tissues everywhere, where certain affinities between oxygen, carbon and hydrogen, are constantly being satisfied, resulting in certain molecular and physical disturbances; these very changes themselves, being the resultants of disturbed equilibrum in forces existing in the various compounds of the organism. No change in matter in any form can take place without a corresponding disturbance, in equilibria of force. For instance, when a molecule of oxygen, which consists of a chromatic scale of atoms (we will say seven, more or less) unites with a molecule of carbon, similarly made up, a disturbance and loss of balance has taken place in these two *so-called* simple elements and new affinities, established by some mutual molecular polar arrangement, by which a certain chemical relationship is brought about, making them into one body instead of two, as before, with a closer approximation of molecules and atoms, than previously existed, by a mean reduction in bulk or volume, *i. e.*, three volumes to two, and a closer approximation of atoms and molecules than previously existed. During this change in the elements the chemical and physical results are just the same as though taking place out of the body, *i. e.*, heat and magnetism are developed, or set free, and if any suitable conductors are present, certain amounts of magnetic force are sent over them, and if no suitable conductors be present, heat and static electricity only are manifested.

It is the collision and clashing of molecules, the rushing together, then recoiling of atoms, (as of men in battle,) the impact and recoil, the evolutions and counter-evolutions of atoms, according to the dictates of their drill master, their law of discipline that must be obeyed, that constitutes chemical change; as Mr. Huxley says of the growing forrest,—if our ears were fine enough to hear the clashing of atoms in vegetable growth, it would be like the roar of a great cataract or battle; it would be deafening to listen to.

During this molecular change, the disturbance results in motion among molecules, and *vis viva* results, and what is called *dead matter*, follows in the form of carbonic acid and water; the carbonic acid serves only to drug the system, if not regularly removed, its presence at the same time conserves another end; that is, to somewhat smother the oxidizing processes, and prevent a too rapid development of heat. Hence we discover that this dead matter is of service in normal quantities during its presence in the body. The same process takes place during the burning of a house: carbonic acid and water quench combustion in or out of the body.

During this process of combustion or oxidation, motion among atoms seems to be the chief effect brought about. This same motion impresses more or less all other bodies of matter in contact with it, whether solid, liquid, or gaseous, the disturbance more or less deranging, or rather rearranging the molecules by interfering with their polarity, which is a universal law of matter.

A certain amount of heat force developed during oxidation, is taken up by the carbonic acid and watery vapor, and rendered static, or *so-called dead matter*, and so remains until the combination in turn be broken up by new affinities, Then again, there is *vis viva* generated, which properly applied, is a working power. In the combustion of a given amount of carbon and hydrogen, whether in the form of incandescent combustion, or by the slower processes in the animal economy, or decay and putrefaction of dead bodies or plants, the same aggregate amount of force or *vis viva* is developed.

When oxidation of zinc takes place, as in the magnetic battery, the larger per cent. of force generated, becomes magnetic force, and if proper conductors be supplied, currents are establishad, certain amounts of heat being generated at the same time, as the solution of zinc becomes heated, and by abstracting the magnetic currents, heat is generated in excess of magnetism. The oxidation of zinc in the battery closely simulates the oxidizing of carbon and hydrogen in the body, where heat and magnetism are established in a similar manner.

In all these processes the same amount of oxygen consumed produces about the same results.

In the animal organization, motion may be regarded as life, and inertia death. All the motions taking place in the body, both molecular change and movement in mass, when in normal amounts, may be taken as physiology; in abnormal amounts, as pathology. Oxidation and nutrition are all the molecule changes taking place in the living body. Oxidation of a molecule of carbon and hydrogen in any tissue, makes room for a molecule of protoplasm, as nutrition, if present, and if not present, too much heat may be generated, and too little magnetism, and if nutrition be too long withheld, fever and death result by starvation, *i. e.*, inanition and syncope. Other causes than the withholding of food, may bring about waste and impaired nutrition, such as fevers in general, when too much oxidation and too little nutrition, then too much heat and too little magnetism result. This wasting process by oxidation is purely chemical just the same in and out of the body, varying only in degree.

The process of nutrition, the sequence of oxidation, is purely a vital process in obeyance of chemical laws, and takes place nowhere outside of living organisms.

This process of waste and reproduction, or life and death of the part, alternately comes under the head of molecular physiology, in contradistinction to other functions and processes; such as the circulation of the blood, respiration, muscular contraction and exertions, and all other processes not dependent on direct, but indirect molecular change.

All the varied changes and processes taking place in the healthy body, are one and all physiological; the digestion and assimilation of food, its entrance to the circulation, and into the various tissues of the body, the uses and distribution made of it, until its final exit again as *debris* or waste matter from the various tissues.

While the kidneys are a set of strainers only, the liver is a great chemical laboratory, having important controlling influences over all other secretions of the entire body, and any derangements here are of vital and signal importance in the category of almost all acute and many chronic diseases. There are other secretions and excretions, as the salivary, pancreatic, pirspiratory, serous and mucous, in general of great importance in the economy.

TO BE CONTINUED.

